# A Refined Prediction Parameter for Molecular Alignability in Stretched Polymers and a New Light-Harvesting Material for AlGaAs Photovoltaics

**DOI:** 10.3390/polym14030532

**Published:** 2022-01-28

**Authors:** Manuel Hohgardt, Franka Elisabeth Gädeke, Lucas Wegener, Peter Jomo Walla

**Affiliations:** Department for Biophysical Chemistry, Institute for Physical and Theoretical Chemistry, Technische Universität Braunschweig, 38106 Braunschweig, Germany; m.hohgardt@tu-braunschweig.de (M.H.); f.gaedeke@tu-braunschweig.de (F.E.G.); lucas.wegener@tu-braunschweig.de (L.W.)

**Keywords:** artificially light-harvesting, luminescent solar concentrators, molecular alignability prediction, redirecting diffuse light

## Abstract

Light-harvesting concentrators have a high potential to make highly efficient but precious energy converters, such as multijunction photovoltaics, more affordable for everyday applications. They collect sunlight, including diffusively scattered light, on large areas and redirect it to much smaller areas of the highly efficiency solar cells. Among the best current concepts are pools of randomly oriented light-collecting donor molecules that transfer all excitons to few aligned acceptors reemitting the light in the direction of the photovoltaics. So far, this system has only been realized for the 350–550 nm wavelength range, suitable for AlGaInP photovoltaics. This was achieved by using acceptor molecules that aligned during mechanical stretching of polymers together with donors, that stay random in that very same material and procedure. However, until recently, very little was known about the factors that are responsible for the alignability of molecules in stretched polymers and therefore it was difficult to find suitable donors and acceptors, as well as for other spectral ranges. Recently, a structural parameter was introduced with a high predictivity for the alignability of molecules that contain rigid band-like structures or linear aromatic π-systems. However, for light concentrators in more red spectral ranges, molecular systems often contain larger and extended, planar-like π-systems for which the previously reported parameter is not directly applicable. Here, we present a refined prediction parameter also suitable for larger plane-like structures. The new parameter depends on the number of in-plane atoms divided by out-of-plane atoms as determined by computational geometry optimization and additionally the planar aspect ratio for molecules that contain only in-plane atoms. With the help of this parameter, we found a new system that can efficiently collect and redirect light for the second 500–700 nm AlGaAs layer of current world-record multijunction photovoltaics. Similarly, as the previously reported system for the blue-green layer, it has also overall absorption and re-directioning quantum efficiencies close to 80–100%. Both layers, together, already cover about 75% of the energy in the solar spectrum.

## 1. Introduction

Solar energy is one of the renewable energy sources with the greatest potential. However, the efficiency of conventional silicon solar cells is limited by the Shockley–Queisser limit, so that a theoretical efficiency of ~30% cannot be exceeded here [1,2]. Therefore, in recent years, work has been carried out on more effective photovoltaic cells and multi-junction solar cells with much higher levels of efficiency have been developed. However, the materials used are very expensive [3,4,5]. One solution to make such a type of photovoltaics usable for more everyday applications would be to collect the light on a large area with more affordable material that redirects it towards much smaller areas of precious high efficiency solar cells. When only direct irradiation of sunlight would reach the earth’s surface, lenses would very easily fulfil this task; however, very often the sunlight is diffusively scattered by clouds or other surfaces and there is no way to refocus the light scattered by standard ray optics. As an alternative, diffusive light can be concentrated by luminescent solar concentrators (LSC) but, unfortunately, no such system was capable to really redirect nearly 100% of the photons onto much smaller photovoltaic areas in the past [6].

If a LSC is used that consists of only one type of pigments, there are high intrinsic losses termed escape-cone or reabsorption losses. The former is due to the fact that light absorbed by molecules is usually reemitted in a similar direction as it came from, instead of being redirected to the photovoltaics. The latter occurs because light is reabsorbed within the waveguide due to the high concentration necessary for absorption of all sunlight. Both lead to losses of energy that are intrinsically higher in single-pigment LSCs than the performance advantage of high efficiency photovoltaics [7,8,9,10,11]. A great deal of research has been carried out in recent years to find LSCs with the necessary better efficiencies [12,13,14,15,16,17,18,19,20,21,22], but so far the necessary level of near to 100% light re-direction quantum yield was not reached.

**Figure 1:**
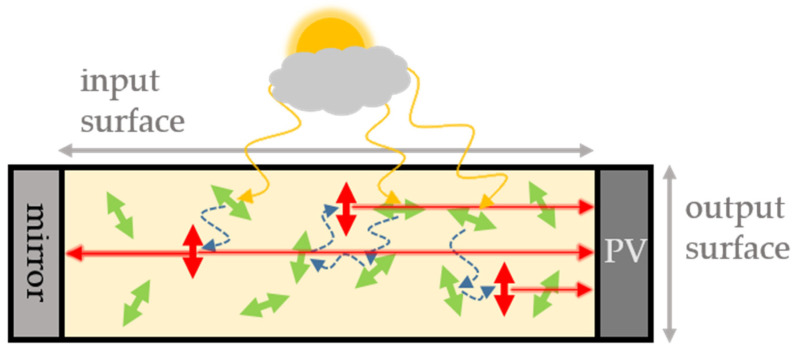
In the FunDiLight LSC concept randomly oriented donor molecule pools (green) absorb diffuse sunlight from all directions (yellow arrows) and transfer the energy (blue arrows) to few aligned acceptor molecules (red). The oriented acceptor molecules then emit specifically in the direction of the photovoltaic (PV, gray). The potential concentration factor is the ratio of input and output surface. The FunDiLight LSC concept avoids intrinsic re-absorption and escape-cone loss mechanisms of previous LSC concepts as it allows for high light absorption with the highly concentrated donor molecules on short optical path lengths (and, consequently, small output surfaces) while avoiding reabsorption due to the low concentrations of the emitting acceptors and escape-cone losses as the oriented acceptors emit almost all light in directions towards the PV. This concept enabled for the first time overall light-re-directioning quantum efficiencies on the order of 90% while still allowing for reasonable high concentration factors (ratio of input surface/output surface): the output surface can be small as all light is absorbed by the highly concentrated donors on short optical path lengths and the input surface can be large as only little light is lost due to reabsorption by the low concentrated, oriented acceptors on the longer path to the output surface.

One intrinsic loss mechanism of conventional LSCs, the high reabsorption losses, can be overcome by donor–acceptor fluorophore systems. This requires, however, that the donors are present in excess in order to collect all sunlight on short optical path lengths that are necessary for a high concentration factor (=input surface/output surface, Figure 1) and transfer it also with near to 100% efficiency to the acceptors. The acceptors, in turn, must be far less concentrated to avoid reabsorption on the necessary long pathway to the photovoltaics, which is also necessary for a high concentration factor. However, even then, the acceptors would emit light in any direction, resulting in losses of those rays, that are not directed into the direction of the PV cell (escape-cone losses).

Therefore, the best system so far is a two-component system that has a larger pool of randomly oriented light-donor molecules and a much lower concentration of acceptors that are all aligned parallel to the photovoltaics (Figure 1). The highly concentrated and randomly oriented donors collect the sunlight from all directions and pass it on to the aligned acceptors via radiationless energy transfer. The aligned receptors, in turn, then radiate specifically in the direction of the PV cell. This minimizes both the escape-cone losses and the losses due to reabsorption in on system, which is also called FunDiLight LSC (funnelling diffuse light re-directioning LSC) [23,24].

Realizing the necessary alignment of the acceptors and the random orientation of the donors in the very same material was possible because, under mechanical stretching of certain polymers, some types of molecules were aligned and others were not in that very same material and procedure. In the first case of a FunDiLight LSC poly(vinyl alcohol) (PVA) was used as a waveguide. However, until recently it was very unclear as to why some molecules are aligned in the polymer during such a procedure whereas others stay random. This knowledge, however, is crucial to developing high-efficiency FunDiLight LSCs for other spectral ranges. Research into the reasons for this found a parameter with a high predictive factor for a molecules alignability in the polymer PVA that depend on the number of atoms that lie within rigid band-like structures of a molecules structure versus the number of atoms that lie outside this band [24]. However, this parameter is only well defined for molecules that contain a single rigid band-like structure. For larger π-systems that are often necessary for light-harvesting in red spectral ranges, and that contain structures that cannot be easily assigned to a single band, this parameter was not well defined. Therefore, it was also more difficult to find appropriate donor and acceptor dyes for highly efficient FunDiLight LSCs in the red spectral range [23,24].

Here, we present a refined and very well-defined parameter that is also capable to predict the molecular alignability of larger π-systems and planar structures in stretched polymers. The new parameter depends on the number of in-plane atoms divided by out-of-plane atoms as determined by computational geometry optimization and additionally the planar aspect ratio for molecules that contain only in-plane atoms. Using this parameter, we found a new FunDiLight LSC that can efficiently collect and redirect light also in the more reddish AlGaAs band gap of current world-record multijunction photovoltaics [3]. Similarly, as the previously reported system for the blue-green layer, it has also an overall absorption and re-directioning quantum efficiency on the order of 90%. Both layers together already cover about 75% of the energy of the solar spectrum.

## 2. Materials and Methods

### 2.1. Sample Preparation for Screening the Alignability of Dyes Containing Larger π-Systems

For screening of the alignability of all dyes investigated in this study, first, 2.2 g poly(vinyl alcohol) (PVA) and the corresponding dye are weighed and dissolved in 20 mL dimethyl sulfoxide, so that a 5 × 10^−4^ M dye-solution is created. This solution is heated for 3 h at 70 °C under a nitrogen atmosphere while stirring. About 4 g are weighed into a Petri plate and dried for 2 days at 60 °C under 200 mbar. The resulting film is stretched 500% so that one can see which molecules are aligned. For the three-dimensional single-molecule polarization measurements a foil with a concentration of 10^−10^ M was prepared.

### 2.2. Sample Preparation of the Red FunDiLight LSC

As described before, 2.2 g PVA and 38.56 mg Lumogen F Red 300 and 1.73 mg Oxazine 170 perchlorate are weighed out and dissolved together in 20 mL dimethyl sulfoxide (DMSO). This solution is also under a nitrogen atmosphere while stirring heated for 3 h to 70 °C. About 4 g are weighed into a Petri plate and dried for 2 days at 60 °C under 200 mbar. The resulting film is stretched by 500%, with the Oxazine 170 aligning and the Lumogen F Red 305 molecules remaining randomly oriented.

### 2.3. Fluorescence Spectroscopy to Dertermine the Alignability

A similar setup has already been described [24]. Briefly, the polymers are placed in the fluorescence spectrometer. In addition, polarization filters are built in so that different combinations of polarizations can be measured. From this, the intensities of the parallel and perpendicular components can be determined. For more details, see [24].

### 2.4. Three-Dimensional Single-Molecule Orientation Microscopy

A similar setup has already been described [24]. This time only, the Coherent Chameleon laser was used, together with an optical parametric oscillator, to set the wavelengths used. The polarization of the laser is rotated by a motorized half-wave plate during the entire measurement. Since light is only absorbed parallel to the transition dipole moment, a modulation of individual dyes can be seen. The dyes are measured from three directions. This is achieved in that two wedge prisms arranged contrary to one another move the laser beam laterally to the optical axis. When the laser hits the objective, it leaves it at an angle of approximately 35° to the optical axis. The fluorescence of the wide field microscope setup is detected with an EMCCD camera. For more details, see [24].

### 2.5. Pump-Probe Spectroscopy

A similar setup has already been described [23,24]. A high repetitive laser system (Coherent OPA/Coherent RegA operated at 120 kHz, pumped by Coherent Verdi) was used for the pump-probe experiments. The pump wavelength was set to 580 nm for all measurements and a BP 580/10 (Thorlabs) was placed before the sample. A linear gradient filter and a miniature spectrometer USB2000+ (Ocean Optics) were used to obtain certain wavelengths from the OPA white light for the probe laser. To improve the signal to noise ratio an ultrafast photodiode (provided by Prof. D. Schwarzer) with a set of 10 bandpass filters for every used wavelength BP600/10-690/10 (Thorlabs) was used directly behind the sample. One half-wave plate (achromatic half-wave plate, 400–800 nm, THORLABS) in each beam-path was used for the polarization dependent measurements. A special sample holder was used to minimize photobleaching by rotating the foil while maintaining its orientation, similar to Pieper et al. [23]. The analysis of the data was completed similarly to in Willich, Wegener et al. [24].

## 3. Results

### 3.1. An Improved Prediction Parameter and Scheme for the Alignability of Molecules in Stretched Poylmers

To characterize the experimental alignability of the molecules investigated here, first fluorescence excitation spectra were recorded with different polarization filters and the ratio in the fluorescence intensities detected with polarization filters parallel or perpendicular to the polymer stretching direction, ∆I_‖_/I_⊥_, was determined, as has already been described in Willich, Wegener et al. [24]. In case of no molecular alignment, ∆I_‖_/I_⊥_ = 1 because the fluorescence emission is the same parallel and perpendicular to mechanical stretching direction. When more molecules are oriented along the stretching direction, the detected intensity in the parallel direction increases, ∆I_‖_/I_⊥_ > 1. The larger ∆I_‖_/I_⊥_, the higher the alignment. Values below 1 do not usually occur or mean an alignment of the transition dipole moments orthogonal to the direction of stretching.

**Figure 2:**
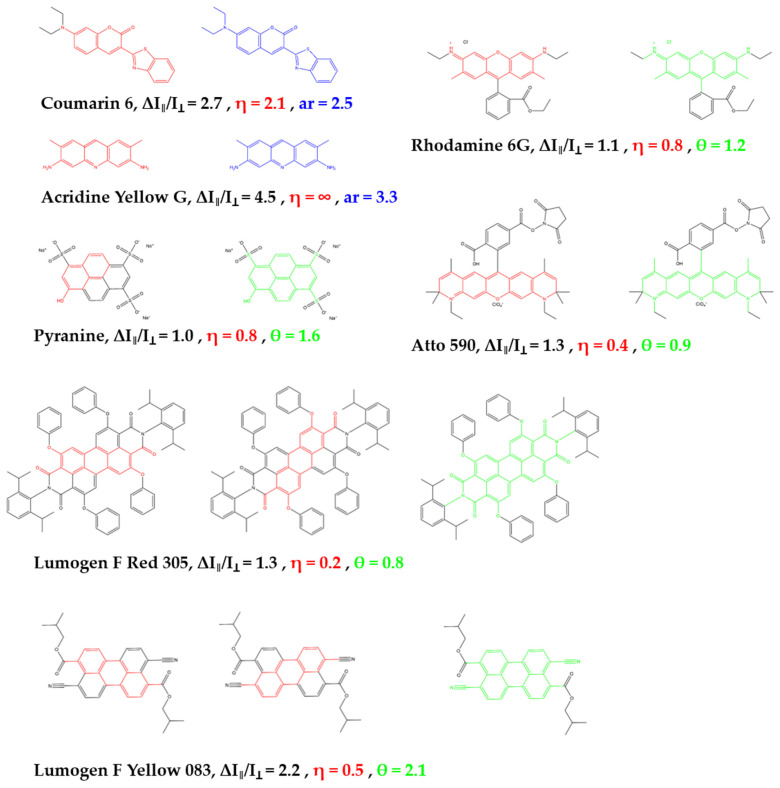
Exemplary structures of molecules denoted with atoms counted for different predictors for the experimentally observed alignability, ∆I_‖_/I_⊥_, in stretched polymers. Green: Atoms counted as in-plane atoms, N_InPlane_, for the predictor θ presented in this work. Red: Atoms countable as intra-band atoms, N_InBande_, for the predictor η presented in [24]. Blue: For molecules in which all atoms lie in one plane, the aspect ratio (ar) serves as robust predictor.

As already mentioned in the introduction, a parameter with a good predictive power for the alignability of organic dyes in mechanically stretched PVA matrix has already been introduced in Willich, Wegener et al. [24]. This parameter, η, was determined as follows. First, the longest rigid and planar band in the molecule was identified. This band can consist of a linear chain of aromatic rings, such as in Acridine Yellow G, or can be also generated by other structural factors, such as in Coumarin 6, in which molecular rotation of a single bond is inhibited by interactions between certain intramolecular groups. In cases that were not immediately clear from the structure itself, a simple computational geometry optimization (Chem3D, minimize energy (as MM2 Calculation)) was used to determine structural parts that are planar, rigid bands. For example, Coumarin 6 is planar throughout the entire band-like structural part denoted by red color in Figure 2. From the structure itself, it was not entirely clear whether steric effects tilt both groups connected by the single bond but the computational geometry optimization confirmed that the red part is planar, likely due to stabilizing interactions between the sulfur and the neighboring nitrogen atom. All the atoms within the plane of this rigid band were then counted as intraband atoms, N_Band_, whereas all atoms outside this rigid band, i.e., flexible side chains or rigid groups that point in different directions than the rigid core band, are counted as out-of-band atoms, N_OutOfBand_ (See, for example, red bands in Figure 2). Hydrogen atoms were simply counted with the assigned atoms they were attached to. The ratio of these atoms,

η = N_Band_/N_OutOfBand_ (1)

had a very high predictive power for the alignability, ∆I_‖_/I_⊥_, of the molecules in stretched polymers. This empiric observation can likely be explained by molecular forces aligning longer rigid bands in polymers during stretching with all atoms pointing out of this band in any three-dimensional directions, N_OutOfBand_, rather hindering this alignment (For more examples see Figure 4 in [24]). Generally, it was observed that an η-parameter of >1 typically predicts a good alignability of ∆I_‖_/I_⊥_ > 1.5.

However, this simple prediction parameter, η, is not well defined for larger molecules with multiple potential bands. For example, in molecules such as Lumogen F Red 305, it is not as straightforward to identify the dominating band, even though η still predicts the low alignability reasonability well, regardless in which structural the dominating band is identified (e.g., Figure 2 shows two possibilities of red band assignments in Lumogen F Red 305).

In order to provide a more precise definition for a prediction parameter also for larger molecules consisting, for example, of more extended π-systems we refined the prediction parameter in the following way. First, we consistently did a geometry optimization for all molecules investigated. Then, all atoms were counted in this optimized geometry that were within the molecular plane of these molecules, N_InPlane_, rather than just those in one band. All other atoms were counted as N_OutOfPlane_. Figure 2 (green) illustrates this count for a couple of molecules. Hydrogen atoms were not included, as they play only a minor steric role but do often occur more dominantly in out of plane groups. This refined parameter

**Figure 3:**
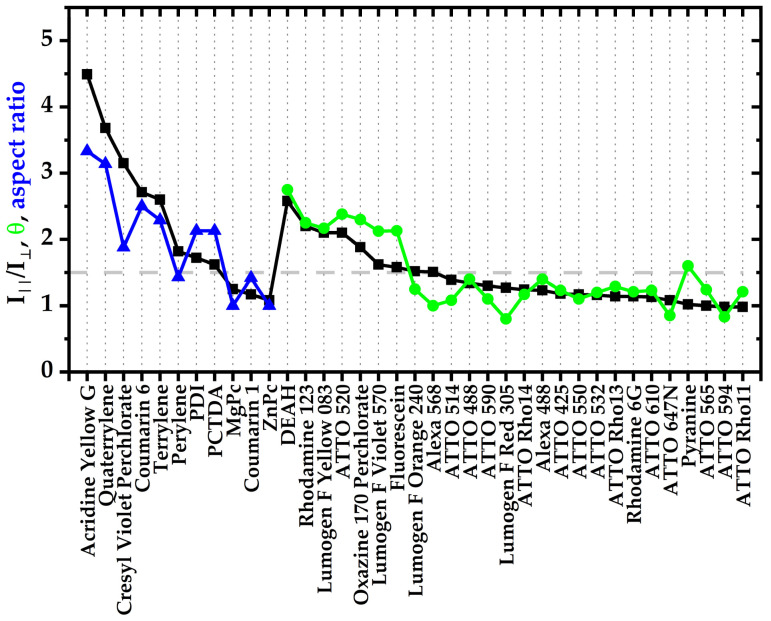
Correlation between the experimentally alignability (black curve, ∆I_‖_/I_⊥_) and the alignability prediction parameter θ (green curve, Equation (2)) and the aspect ratio (blue curve, Equation (3)) as predictor for entirely planar molecules according to molecular geometry optimization calculations.

θ = N_InPlane_/N_OutOfPlane_ (2)

provides similarly well estimates for the alignability as our previous parameter η, (compare green θ values in Figure 3 with experimentally observed alignabilities ∆I_‖_/I_⊥_ in black) but is unambiguous for more planar molecular structures.

In Lumogen F Red 305, for example, the total number of atoms in the molecule is 82 without hydrogens. There are 36 atoms inside a plane and 46 outside, which results in θ = 0.8 (Figure 2), and corresponds well with the experimental observation of little alignability. Another important example is Lumogen F Yellow 083. This is also a molecule with a larger π-system that does not easily allow to identify the largest rigid band. Indeed, the rigid-band bases parameter η = 0.5 does not predict any alignment. However, the new parameter presented here is precisely defined also for such molecules and predicts with a value of θ = 2.1 very well the experimentally observed alignability of ∆I_‖_/I_⊥_ ~ 2.2.

**Figure 4:**
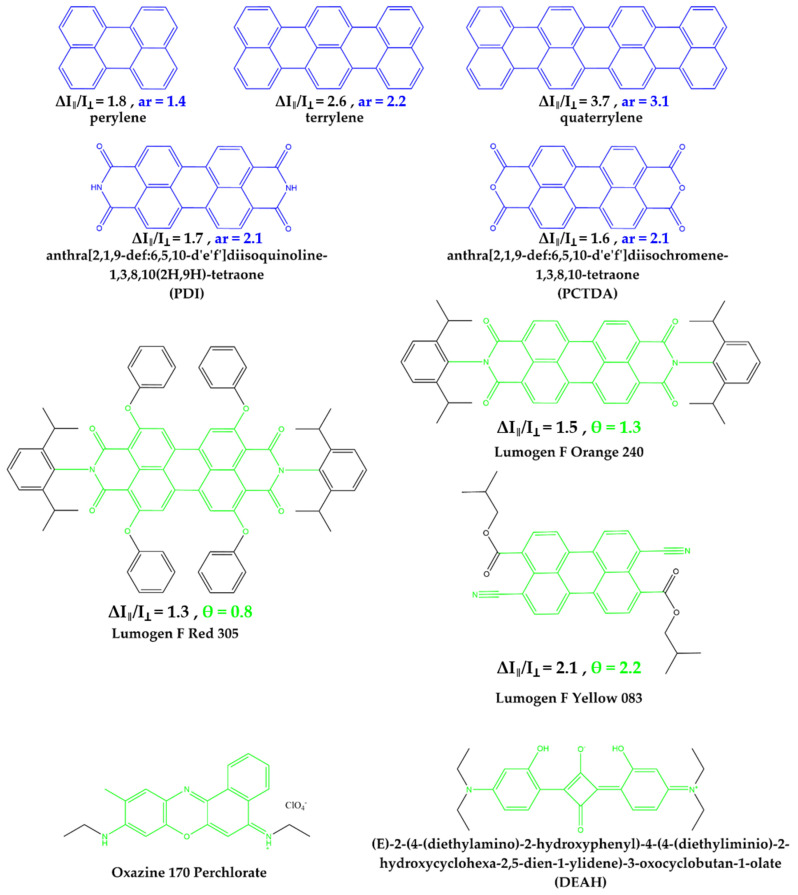
Structures of rylenes and rylene derivates exemplary for planar molecular structures that either do or do not contain any out-of-plane atoms. When there was no out-of-plane atom (blue structures) the aspect ratio (Equation (3)) is a good predictor for the experimentally observed alignabilities, ∆I_‖_/I_⊥_. Otherwise, the θ–parameter (Equation (2)), that is determined from the ratio of in-plane atom (green) number over the number of out-of-plane atoms (black), is a good predictor.

**Figure 5:**
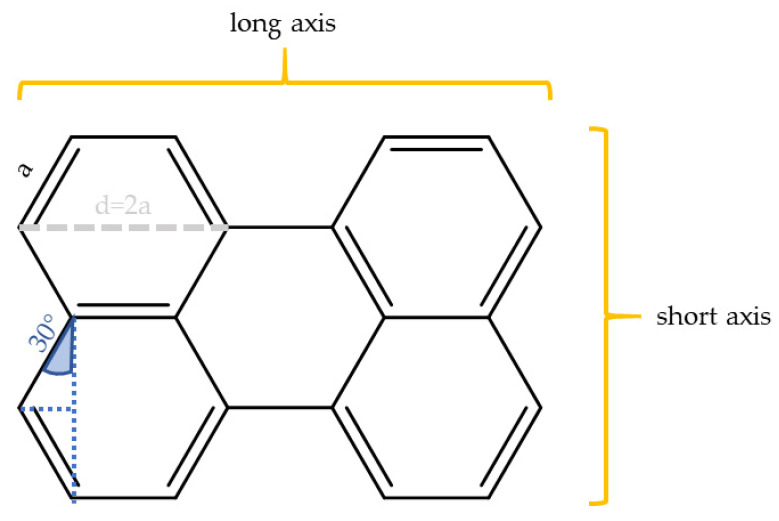
In planar molecules that do not contain any out-of-plane atoms the aspect ratio is a good predictor for the alignability in stretched polymers. The figures show exemplarily how the aspect ratio can be determined from the molecular structure of perylene. For details see text.

In cases, however, in which *all* atoms are in plane according to geometry optimization, N_OutOfPlane_ becomes zero and Equation (2) is not defined anymore. Important examples are rylenes, such as perylene, terrylene, and quaterrylene (structures are shown in Figure 4). Still, better alignment was observed experimentally for structures that resemble rigid band like structures, similar as our previous empirical observation that led to the band-based prediction parameter η, (Equation (1)). To account for this observation, we considered the aspect ratio for such completely planar molecules that do not contain any out-of-plane atoms, N_OutOfPlane_ = 0. To do so, the rigid planar parts of the molecules were first simplified as simple geometric shapes (e.g., benzene ≡ hexagon) with all bonds assumed to be of approximately equal length. With this simplification, the length of the long and short axis of the rigid, planar part of the molecules was then computed in units of the length of one bond, a. Figure 5 illustrates this exemplarily for perylene. Due to geometric consideration the aspect ratio, ar, is in this case

ar = l_longaxis_/l_shortaxis_ = 5a/(4a × cos(30°)) = 1.44 (3)

A comparison of the aspect ratio-based parameter computed for these molecules (blue in Figure 3) demonstrates also a high predictive power for the alignability of such molecules in polymers (black curve in Figure 3).

**Figure 6:**
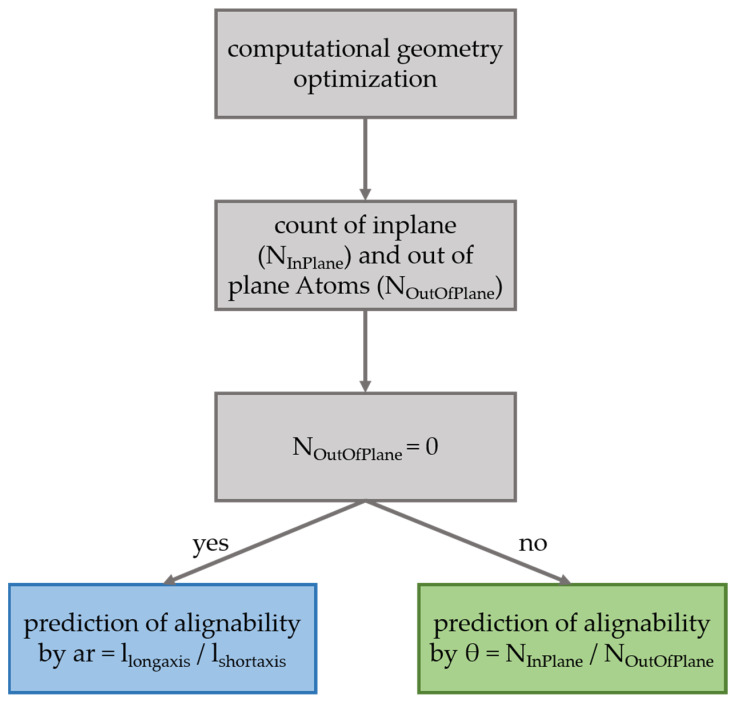
The diagram shows how to estimate the alignability of a molecules transition dipole moment in stretched polymers (PVA) with either the predictor parameter θ (Equation (2)) or the aspect ratio for case of entirely planar molecules (Equation (3)).

The general prediction scheme for the alignability of molecules in polymers is illustrated in Figure 6. First a simple geometry optimization is performed to identify all atoms, expect hydrogens, that are either within the molecules plane, N_InPlane_, or outside the molecular plan, N_OutOfPlane_. If N_OutOfPlane_ ≠ 0, the alignability can be predicted by the ratio defined in Equation (2) (green data in Figure 3). If N_OutOfPlane_ = 0, the alignability can be predicted by the aspect ratio of the plane defined in Equation (3) (Figure 2 and blue data in Figure 3). These predictions correspond very well with the experimentally observed alignabilities, ∆I_‖_/I_⊥_ (black data in Figure 3).

An experimental observation that has so far not been considered in the proposed prediction parameters is the influence of chemical polarization on the alignability. An interesting comparison is that of the molecules PCTDA, PDI and terrylene that all have similar spatial dimensions but are decreasingly polar (structures are shown in Figure 4). All molecules show an alignment but it is noticeable that the ∆I_‖_/I_⊥_ value decreases with greater polarity. The value of ∆I_‖_/I_⊥_ for PCTDA is only half as high as that for terrylene. We suspect that the polar molecules are wedged between polymer chains due to hydrogen bonds, while the non-polar molecules are well placed between the chains using the weaker Van der Waals forces. Therefore, when using the aspect ratio to predict alignability, it must be considered that the polarity has an additional influence.

### 3.2. A New Light-Harvesting Solar Concentrator for the AlGaAs Layer of High-Efficiency Photovoltaics

**Figure 7:**
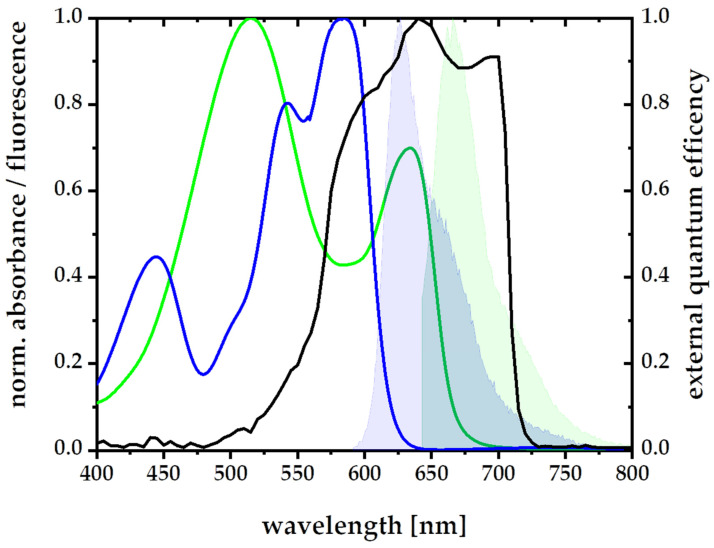
Absorption and emission spectra of Oxazine 170 (green) and Lumogen F Red 305 (blue), respectively. The fluorescence spectrum of Oxazine 170 (green, filled) fits perfectly the EQE spectrum and thus band gap of AlGaAs cells (black) (Data from [3]). The fluorescence Lumogen F Red 305 (filled in blue) overlaps perfectly with the absorption spectrum of Oxazine 170 (green).

So far, high efficiency light-harvesting materials based on the scheme shown in Figure 1 have only been demonstrated for the blue AlGaInP layer of current high efficiency photovoltaics. High efficiency light-harvesting materials for the next, AlGaAs layer, of such high efficiency photovoltaics have not been reported so far, partly due to the above-described difficulties in the ability of predicting the alignability of larger light-harvesting donors and light-redirecting acceptors, that shall either stay randomly oriented or align in that same material during stretching. In order to build a FunDiLight-LSC for the second band gap of the currently best solar cell by Geisz et al. now a suitable donor or acceptor can be taken from Figure 3 [3]. Lumogen F Red 305 is ideal as a donor. No alignability (θ = 0.8) is predicted which is also confirmed experimentally (∆I_‖_/I_⊥_~1.3). In addition, the spectral requirements fit (Figure 7) and a high fluorescence quantum yield of almost 100% suggests a very good energy transfer [25].

In order to find a suitable acceptor, the steric alignment requirements for the molecule must be met in addition to spectral requirements for effective energy transfer and photovoltaics bandgap match, as well as highest possible fluorescence quantum yields. These requirements are met, among others, by the squaraine dye DEAH and the dye Oxazine 170. These two are predicted to be alignable by θ-parameters of θ = 2.75 (DEAH) and θ = 2.3 (Oxazine 170), and is confirmed by the experimentally measurement alignability. Even though squaraine dye has a better fluorescence quantum yield of 86% compared to the Oxazine with 63% in solution, it photodegraded very quickly. Therefore, Oxazine 170 was selected as the acceptor. In addition, it is also observed very often that the fluorescence quantum yields in solid environments, such as polymers, are significantly larger than in aqueous solution. To generate a high-efficiency light harvesting system with Lumogen F Red 305 ^®^ and Oxazine 170, we first calculated optimized donor/acceptor ratios as well as concentrations using our previously published computational ray tracing tool [26] and improved the overall quantum efficiencies experimentally, thereafter. The following characterization of the systems performance was similarly completed as reported previously [27,28].

**Figure 8:**
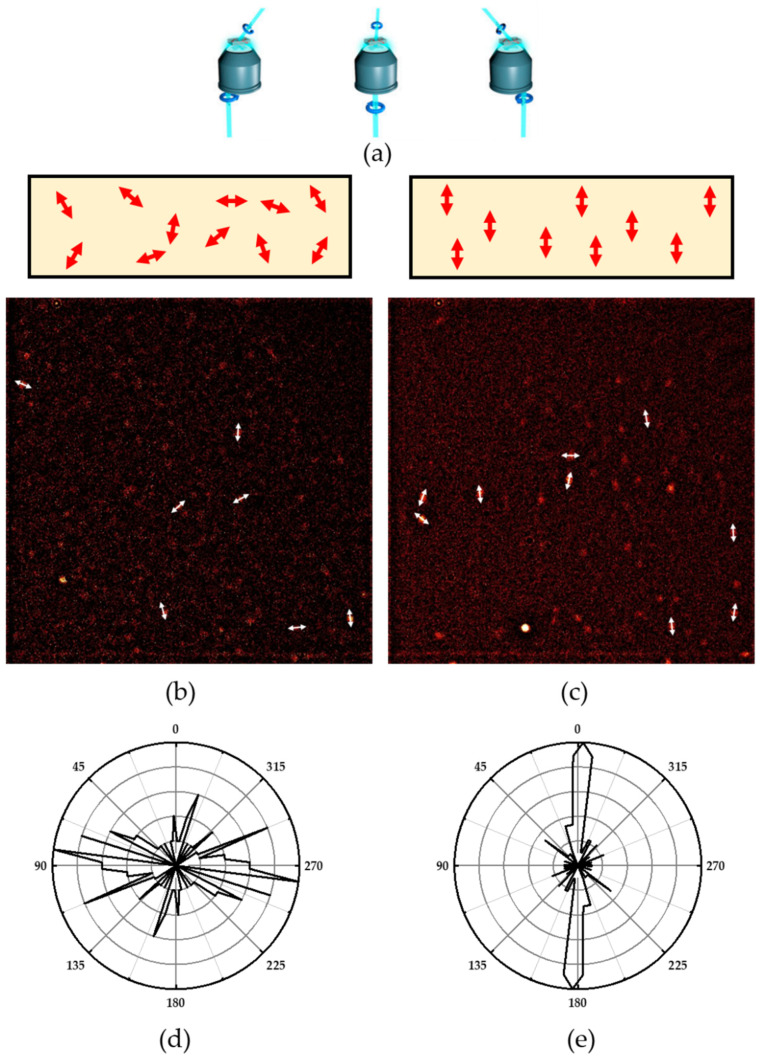
In 3D single molecule orientation microscopy, the sample is illuminated from three different directions with rotating polarization (**a**). The analysis of the observed fluorescence intensity modulations allows to determine the 3D orientation of single molecules in the unstretched (**b**) and stretched polymer (**c**). Polar plots of histograms of the azimuth angles show a clear alignment along the polymer stretching direction (**e**) that is not present in the unstretched control (**d**).

First, the angle distribution of aligned Oxazine 170 was examined in a single molecule 3D orientation microscope. In such a microscope, the stretched or unstretched polymer containing Oxazine 170 is illuminated from three different directions (Figure 8a) while the polarization of the light is rotated. Since light is best absorbed with a polarization vector parallel to the transition dipole moment of the fluorophore, differently oriented dyes are excited at different times. Therefore, the fluorescence traces of the single dyes also show modulation. The 3D orientation of each individual Oxazine 170 molecule can then be determined from these modulations observed from the three different directions. Figure 8b,c shows a typical microscope image from individual Oxazine 170 molecules in PVA. The angular distribution of the flat azimuth angles is shown in polar plot histograms in Figure 8d,e.

Although a very random distribution can be seen in unstretched polymers in Figure 8d, in Figure 8e there is a clear majority of molecules aligned in the stretched polymer.

**Figure 9:**
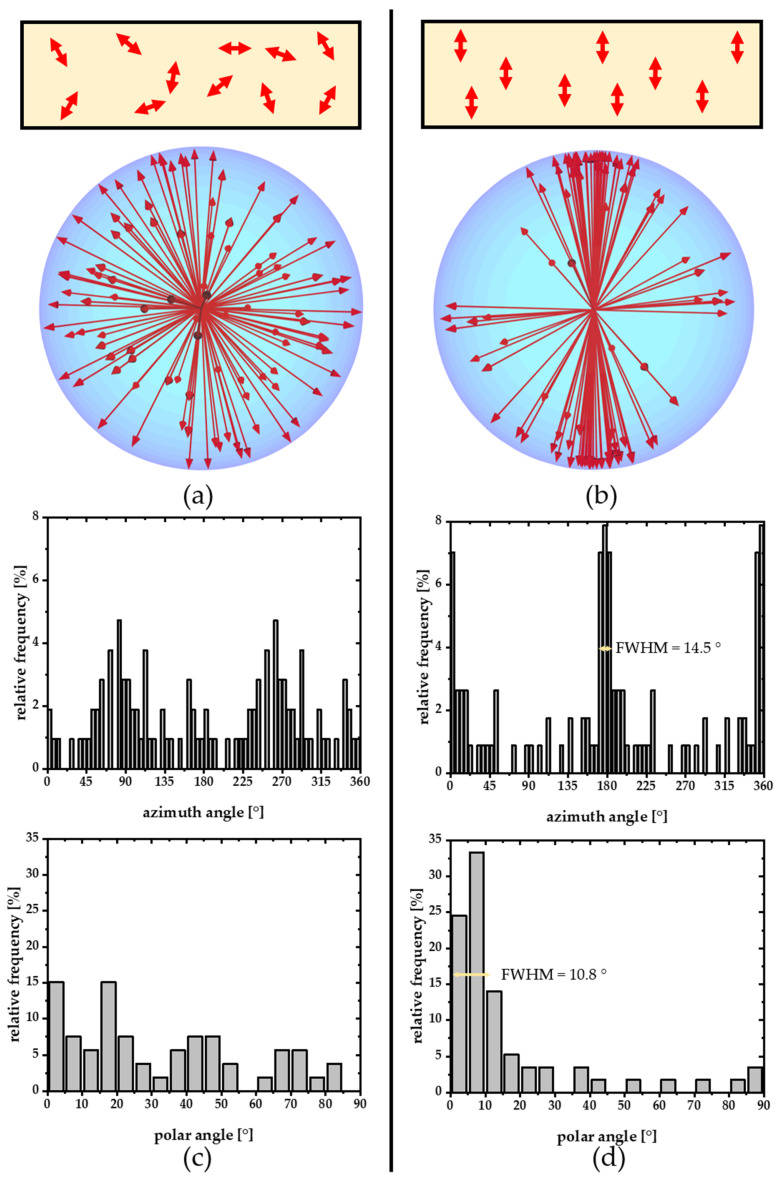
Detailed representation of the 3D angular distribution of the single molecules in an unstretched (**a**) and stretched polymers (**b**) as spherical plot, along with the corresponding azimuth (**b**,**c**) and polar angle distributions (**c**,**d**). Note that the measured polar angle distribution has always a tendency to overrepresent flat angles, even in random orientation distributions (**c**) as upright molecules emit most of their light perpendicular to the microscope objective and are, therefore, hard to detect.

Figure 9 shows a more detailed representation of all single molecules investigated along with linear presentations of the azimuth and polar angle histograms. After stretching, more than half of all molecules lie flat in the plane within 10°. In addition, over half of the molecules are approximately 20° around the direction of stretching. Gaussian fits show a half-width of the azimuth angle distribution of 14.5° and a half-width of 10.8° for the polar angles (Figure 9d).

Based on the microscopic data, the orientation of the acceptor could be reliably verified. This is essential for a precise FunDiLight LSC. However, efficient and fast energy transfer is at least as important for overall high light-re-directioning quantum efficiencies.

Therefore, pump-probe measurements were completed for the donor–acceptor system to investigate the dynamics of energy migration and dipole reorientation in more detail.

**Figure 10:**
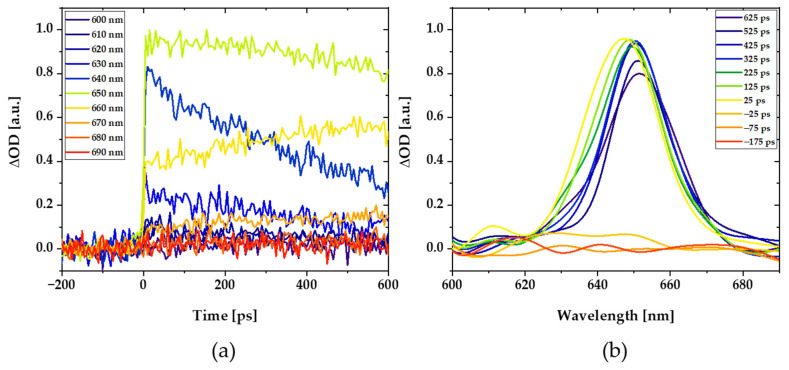
Pump-probe data of the unstretched donor–acceptor polymer samples observed after pumping the donors at λ_exc_ = 590 nm and detecting the acceptor kinetics at various probe wavelengths (**a**) as well as the transient pump-probe-spectra converted therefrom (**b**).

First, a spectrum with different probe wavelengths was recorded after pumping the donor at its absorption maximum, λ_exc_ = 590 nm. The highest signal was obtained at a wavelength of λ_det_ = 650 nm (Figure 10a,b), corresponding well with the acceptor absorption and thus arising very likely from acceptor ground state bleaching after receiving energy from the donors. During the temporal evolution, a decrease in the signal can be seen at bluer wavelengths, which is likely indicative of intramolecular vibrational relaxation processes in the acceptor after receiving the energy from the donors in higher vibrational acceptor states. At red wavelengths, an increase can be seen after this first quick step. (This suggests that donors continue to deliver energy to acceptors).

**Figure 11:**
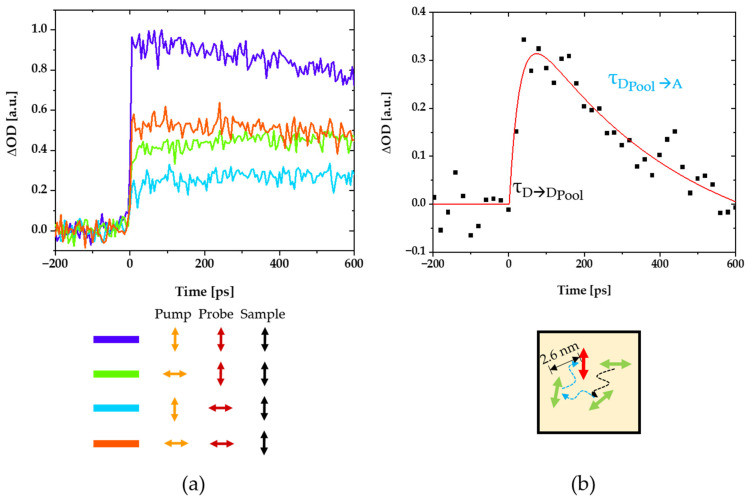
Polarization-dependent pump-probe measurements of the stretched sample with various pump and probe polarizations parallel or perpendicular to the stretching direction (**a**). The time constants for the energy migration and dipole-reorientation dynamics within the donor pool and the subsequent transfer to the acceptors can be determined through the biexponential fit of the difference spectrum of the violet and green curve (**a**) from the stretched sample (**b**). For details of this analysis see text and [24].

Kinetic signals where either fitted by mono- or biexponential rise terms, depending on which better described the observed signals. Since the donor molecules are at different distances from each other and from the acceptor, different time scales, and kinetics are expected. Before the donors close to the acceptor molecules transfer their energy, excitation energy migration and transition dipole reorientation occurs in the larger donor pools. To dissect these processes, polarization-dependent pump-probe measurements were also carried out (Figure 11a). Four different polarizations were measured, with combinations of pump and probe polarizations parallel or perpendicular to the direction of stretching of the polymers. The rise time of these curves reflect direct energy transfer from the (closest) donors to the acceptors and is with about 6 ps is comparable to that what one expects from Förster Theory for a single donor to acceptor transfer at the closest distance of about 2.6 nm between the pigments. With donor pool pump polarization perpendicular to the acceptor probe polarization (and stretching direction), additional kinetic rising components were observed (green in Figure 11a) that are not visible when directly pumping and probing parallel to the stretching direction (violet in Figure 11a). This is due to the additional time necessary to rotate the initial perpendicular transition dipole orientations into transition dipole orientations parallel to the acceptors during energy migration from the donors to the acceptors. A difference spectrum can be formed from these two measurements and a biexponential function can be fitted to this difference spectrum (Figure 11b). The biexponential rise term gives a time constant for the intra donor-pool energy migration and dipole moment reorientation on the order of ~27 ps, as well as a decay time constant of approximately 400 ps, after which the energy transfer from the donor-pool to the acceptors is completed. Overall, the times are all well below the ns lifetime of the donor (7.9 ns [29]), which is why an almost perfect energy transfer efficiency c with a quantum efficiency close to unity can be assumed. In addition, the significantly lower amplitude with perpendicular polarization of the pump and probe beam to the stretching direction compared to corresponding parallel pump and probe beam polarization data once more confirm the acceptor alignment parallel to the polymer stretching directions (Figure 11a red and violet curves).

## 4. Discussion

With the present study, structural factors that lead to an alignability of molecules in polymers—at least in PVA—become clearer. Key factors are obviously the size of rigid planar parts in a molecules structure, as well as the aspect ratio of this plane and the size and number of structural groups that point outside this plane and/or that are flexible. Based on these observations, we provide a refined prediction parameter, θ, and scheme (Figure 6) that is based on the ratio in the numbers of in-plane and out-of-plane atoms observed in a simple geometry optimization calculation (Equation (2), green in Figure 2 and Figure 4)) and the aspect ratio for planar molecules (Equation (3), blue in Figure 2 and Figure 4), that do not contain any out-of-plan atoms at all. These parameters and the scheme predict the alignability (e.g., for light re-directing acceptor molecules, red in Figure 1) or non-alignability (e.g., for randomly oriented light harvesting donor molecules in the same material, green in Figure 1) at least as good as our previously reported parameter, but allows to better predict the alignability of larger molecules (Figure 3). In addition, we found an indication that the alignability decreases with greater polarity. We suspect that this is due to increasing distorting interactions with polar groups of PVA during the stretching. However, this observation is not included in our alignability estimation parameters yet, as it needs more experimental verification.

**Figure 12:**
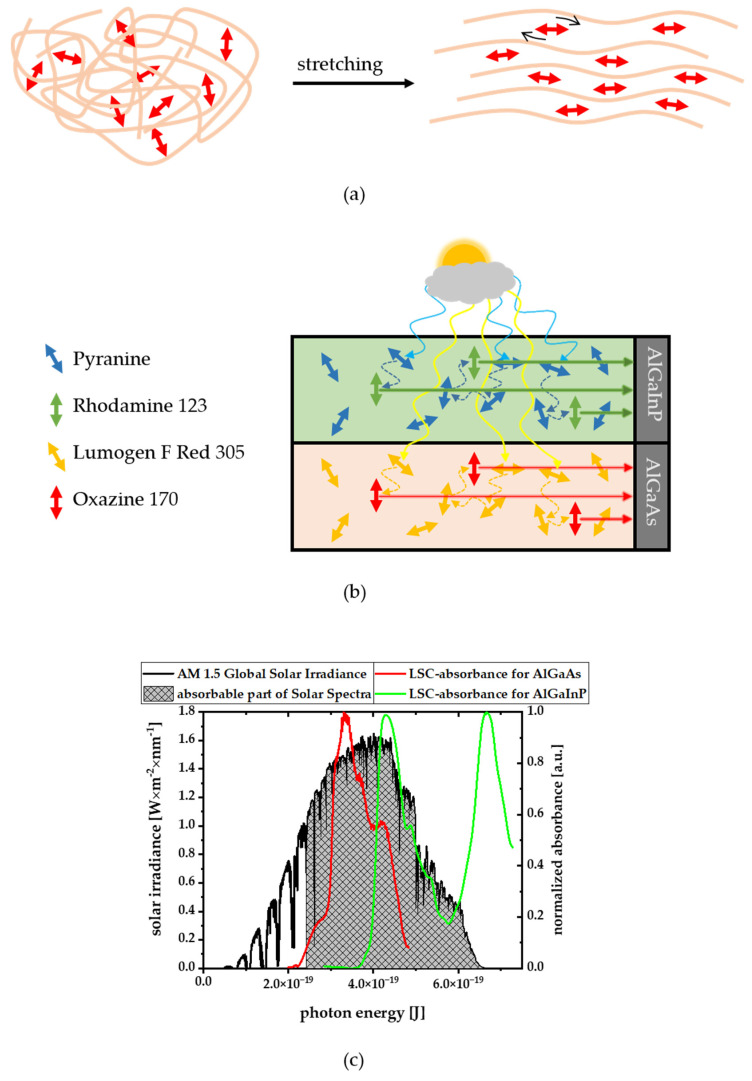
Polymer chains (beige) are likely ordered by the stretching procedure. We suspect that shear forces (small black arrows) can align the dyes (red arrows) between the polymer chains when they have the corresponding structural requirements (see Figure 2, Figure 3, Figure 4, Figure 5 and Figure 6) (**a**). In the envisioned two-layer light-harvesting and energy conversion system light in the wavelength range between 275 and 500 nm is first collected in the upper layer by the randomly oriented Pyranine donor molecules (blue) and transferred to the Rhodamine 123 acceptors (green) aligned parallel to the AlGaInP photovoltaics (grey). Wavelengths longer than 500 nm pass through the first layer and are then absorbed by the Lumogen F Red 305 donors (yellow) and transferred on to the aligned Oxazine 170 acceptors (red). These emit correspondingly in the direction on the AlGaAs PV cell material. (**b**) The part of the solar spectrum ([31,32], Data from [32]) that are entirely harvested by the Pyranine and Rhodamine 123 molecules (green absorption spectrum) and Lumogen F Red 305 and Oxazine 170 molecules (red absorption spectrum) of such a two-layer system is marked by grey color and corresponds already to about 75% of the total solar irradiation energy (**c**).

Obviously, the alignability is a largely steric phenomenon. We find that small hydrogen atoms can be neglected for a good alignment prediction. We found experimental alignment when the θ-parameter was greater than 1.5 or in other words when 1.5 times more atoms are in the plane than outside of it. We suspect that the polymer chains start to orient in one preferred direction when they are mechanically stretched and that the molecules between the ordered chains are aligned by shear forces (Figure 12a). Molecules with larger numbers of rigid or non-rigid groups and atoms pointing outside this plane are more likely to wedge between the chains, hindering alignment in that same direction. We also suspect that this more likely, when more heteroatoms are present that make the molecules more polar. Since the polymer PVA is already polar itself, the wedging of the molecules could be increased during the polymer stretching.

In the case of completely flat molecules, the aspect ratio is also important. We suspect that while all flat molecules align themselves, a rotation around the transverse axis of the molecule allows also for transition emission dipole moments perpendicular to the stretching direction, and, therefore, no alignment of the light emission is observed. With elongated molecules of higher aspect ratios, such rotation becomes less likely or in other words the molecules dipole moment does align in just one direction parallel to the stretching direction. We found that emission dipole moment alignment can be typically found when the aspect ratio was approximately higher than 1.5.

With these insights we were able to seek suitable molecules that act as randomly oriented light-harvesting donor pools (green in Figure 1) and light redirecting acceptor molecules (red in Figure 1) also for other spectral ranges than our previously published system for the blue-green AlGaInP spectral range of high-efficiency photovoltaic [23,24]. Before the present study, this was difficult as the necessary alignment or non-alignment of larger molecules in the very same polymer and stretching procedure could not be predicted as easily with our previous prediction parameter, η, that rather relied on the size of rigid band size structure in smaller pigment molecules.

For the more reddish spectral range necessary for the AlGaAs layer of high efficiency photovoltaics, the relatively flat and long dye Oxazine 170 was found as a suitable example of a fluorophore that aligns very well in polymers when mechanically stretched. It has a prediction parameter of θ = 2.3 and indeed showed an experimental alignability of ∆I_‖_/I_⊥_ ~ 1.9. Together with the bulky Lumogen F Red 305, that stays with θ = 0.8 and ∆I_‖_/I_⊥_ ~ 1.3 randomly oriented in the very same polymer and stretching procedure, it forms a FunDiLight system as illustrated in Figure 1 for the spectral range of the AlGaAs layer. The Donor Lumogen F Red 305 collects exactly the spectral range of the light not covered by the previously published blue layer, and can pass it on to the acceptor Oxazine 170 with high yield, due to perfect spectral overlap. The aligned acceptor can purposefully redirect the energy and the emission of Oxazine 170 fits perfectly with the AlGaAs band gap of high efficiency photovoltaics. Microscopic 3D single molecule orientation measurements confirmed that the light re-directioning acceptors in this system are very well aligned, with 85% of the dyes within 25° of the stretching direction.

The highly efficient light-harvesting donor to light-redirecting acceptor energy transfer is confirmed by efficient, ultrafast energy transfer unveiled by polarized pump-probe spectroscopy (Figure 10 and Figure 11). These experiments also provided valuable insights into the donor pool energy migration and emission dipole moment reorientation on timescales on the order of approximately 6–400 ps. The excitation energy is transferred from the primarily excited light harvesting donors to the donor-pool on a timescale of about 27 ps. From there, the energy is gradually passed on to the acceptors. The final ultrafast one-step donor to acceptor energy transfer step from the nearest donor in the light-harvesting pool to the light-redirecting acceptors time occurs in about 6 ps. Even if the transition dipole moments of the excited donors are very different for the light-redirecting acceptors, they still transferred efficiently all excitons after about 400 ps.

## 5. Conclusions and Perspective

In summary, these results confirm that about 99.9% of light in the 420–660 nm spectral range of the light-harvesting donor is collected in a single foil of 50 µm thickness, at least 98% of these excitations is transferred to the light-redirecting acceptors, and that the acceptors emit about 80% of the light in directions suitable for effective total internal reflection waveguiding to, for example, high-efficiency photovoltaics. The percentage of absorbed light was inferred from the absorption spectrum of a single foil in the spectral range of 420–660 nm (Figure 7), the efficiency of the energy transfer from a direct comparison of the donor fluorescence intensity in the presence and the absence of acceptors [30], as well as the observed ultrafast energy transfer on timescales from 6 to 400 ps (Figure 10 and Figure 11) in comparison to the donor lifetime (7.9 ns [29]) and the emission angle range from the 3D orientation single molecule experiments (Figure 8 and Figure 9) in a similar manner as described in Pieper et al. and Willich, Wegener et al. [23,24]. Therefore, the overall efficiency of the new funneling light-harvesting system for the reddish AlGaAs layer of high-efficiency photovoltaics is as similarly high as our previously reported systems for the blue-green AlGaInP layer [23,24]. Together with the previously proposed system for the blue-green spectral range, these two layers already cover about 75% of the total energy of the solar light (denoted by grey color in Figure 12c).

For the future development we envision two layers of our high-efficiency light-harvesting systems together with the corresponding two layers of high efficiency photovoltaics. This principle is shown in Figure 12b. One of our future aims is to realize such a real word system including all components that are necessary for a high efficiency light harvesting together with high efficiency photovoltaics and a concentration factor (input surface/output surface, see Figure 1) as large as possible and the very little loss mechanisms provided by our FunDiLight approach.

## 6. Patents

The University of Braunschweig (P.J.W.) filed a patent for parts of this work.

## Data Availability

The data presented in this study are available on request from the corresponding author. All study data are included in the article.
